# Chlorpromazine directly inhibits Kv1.3 channels by facilitating the inactivation of channels

**DOI:** 10.1186/s13041-025-01211-z

**Published:** 2025-05-08

**Authors:** Seo-In Park, Soobeen Hwang, Young Lee, Hee-Yoon Lee, Soohyun Kim, Junseo Hong, Su-Hyun Jo, Se-Young Choi

**Affiliations:** 1 Department of Physiology, Dental Research Institute, Seoul National University School of Dentistry, Seoul, 03080 Republic of Korea; 2 Department of Physiology, Kangwon National University School of Medicine, Chuncheon, 24341 Republic of Korea

**Keywords:** Chlorpromazine, Kv1.3 channel, Inactivation, Microglia, Neuroinflammation

## Abstract

Kv1.3 channels in microglia are pivotal in regulating neuroinflammation. The antipsychotic chlorpromazine (CPZ) demonstrates anti-inflammatory effects by decreasing Kv1.3 activity in mPFC microglia. However, the precise mechanism of CPZ’s effect in the mPFC remains unclear, given that CPZ is known to inhibit dopamine receptors and the mPFC contains various cell types with dopamine receptors. In this study, we investigate how CPZ inhibits Kv1.3 channels using human Kv1.3 channel-expressing *Xenopus laevis* oocytes. CPZ directly inhibits Kv1.3 channel currents in a concentration-dependent manner. The CPZ-mediated Kv1.3 channel inhibition is not voltage-dependent, and CPZ accelerates Kv1.3 channel inactivation without significantly affecting its activation. Our findings suggest that CPZ directly blocks Kv1.3 channels without involving other ion channels or receptors, including dopamine receptors, thereby contributing to the understanding of its neuroinflammation-suppressing mechanism.

**Supplementary Information:**

The online version contains supplementary material available at 10.1186/s13041-025-01211-z.

## Introduction

Microglia are non-neuronal immune cells residing in the central nervous system (CNS), tasked with responding to neuroinflammation [1, 2]. In their quiescent state, they survey the CNS for infection and primarily perform a neuroprotective role [3]. However, when activated by external pathogens or acute brain injury, microglia release proinflammatory cytokines, initiating neuroinflammatory pathways [1, 4]. These pathways alter neuronal functions such as neuronal excitability, synaptogenesis, synaptic plasticity, and cognitive functions [5, 6]. Clinically, it is widely recognized that various neurological and psychiatric diseases, including Alzheimer’s disease, autism, and depression, are associated with neuroinflammation [3, 7, 8].

Ion channels expressed in microglia play a central role in modulating neuroinflammation, regulating microglial ramification, surveillance, chemotaxis, phagocytosis, ROS production, and cytokine release [9]. Specifically, the Kv1.3 channel in microglia is deeply involved in neuroinflammation and the pathology of neurological and psychiatric diseases [1, 10, 11]. Additionally, Kv1.3 channels are expressed in dendritic cells [12] and T cells [13, 14] outside the CNS, playing a crucial role in regulating immune functions in the peripheral system. LPS-induced systemic inflammation increases Kv1.3 expression in brain microglia [15]. Conversely, inhibiting the Kv1.3 channel reduces LPS-induced proinflammatory cytokine secretion, suggesting that LPS drives inflammatory responses through Kv1.3 channels [16, 17]. Kv1.3 staining is observed on activated microglia in rodent and human infarct tissue, and the Kv1.3 channel inhibitor PAP-1 reduces tissue damage [18]. Mechanistically, a Kv1.3 channel-dependent pathway is proposed to regulate MHCI-mediated antigen presentation by microglia to CD8 + T cells [19].

Chlorpromazine (CPZ) is a typical antipsychotic drug used to treat schizophrenia and acts as a dopamine D2 receptor (D2R) inhibitor in neurons [20–24]. Interestingly, CPZ and other antipsychotics exhibit anti-inflammatory effects, such as inhibiting LPS-induced production of proinflammatory cytokines [25–27], though the precise mechanism remains unclear [28]. Recently, CPZ has been reported to effectively suppress LPS-induced inflammation, while sulpiride, another D2R-specific antagonist, does not demonstrate efficacy in mitigating LPS-induced inflammation in mPFC microglia [29]. These findings suggest that CPZ may act on additional targets to regulate inflammation. CPZ was found to decrease Kv1.3 currents in rat megakaryocytes [30] and mPFC microglia [29]. However, prior research conducted on brain slices faced limitations in confirming Kv1.3 inhibition due to the coexistence of dopamine D2 receptors and other possible CPZ targets. Thus, the mechanism by which CPZ influences Kv1.3 channels in microglia remains unclear, as it is uncertain whether the effect is direct or indirectly mediated through downstream of other channel- or receptor-related mechanism.

This study aims to address these gaps by elucidating the direct inhibition of Kv1.3 channels by CPZ and detailing its underlying mechanisms. Utilizing a Kv1.3-expressing *Xenopus* oocyte model, we explored the biophysical mechanisms modulating Kv1.3 channel activity without interference from other ion channels. Our findings indicate that CPZ directly blocks the Kv1.3 channel in a masking-block manner.

## Results

### Direct inhibition of human Kv1.3 channel currents by CPZ

**Fig. 1:**
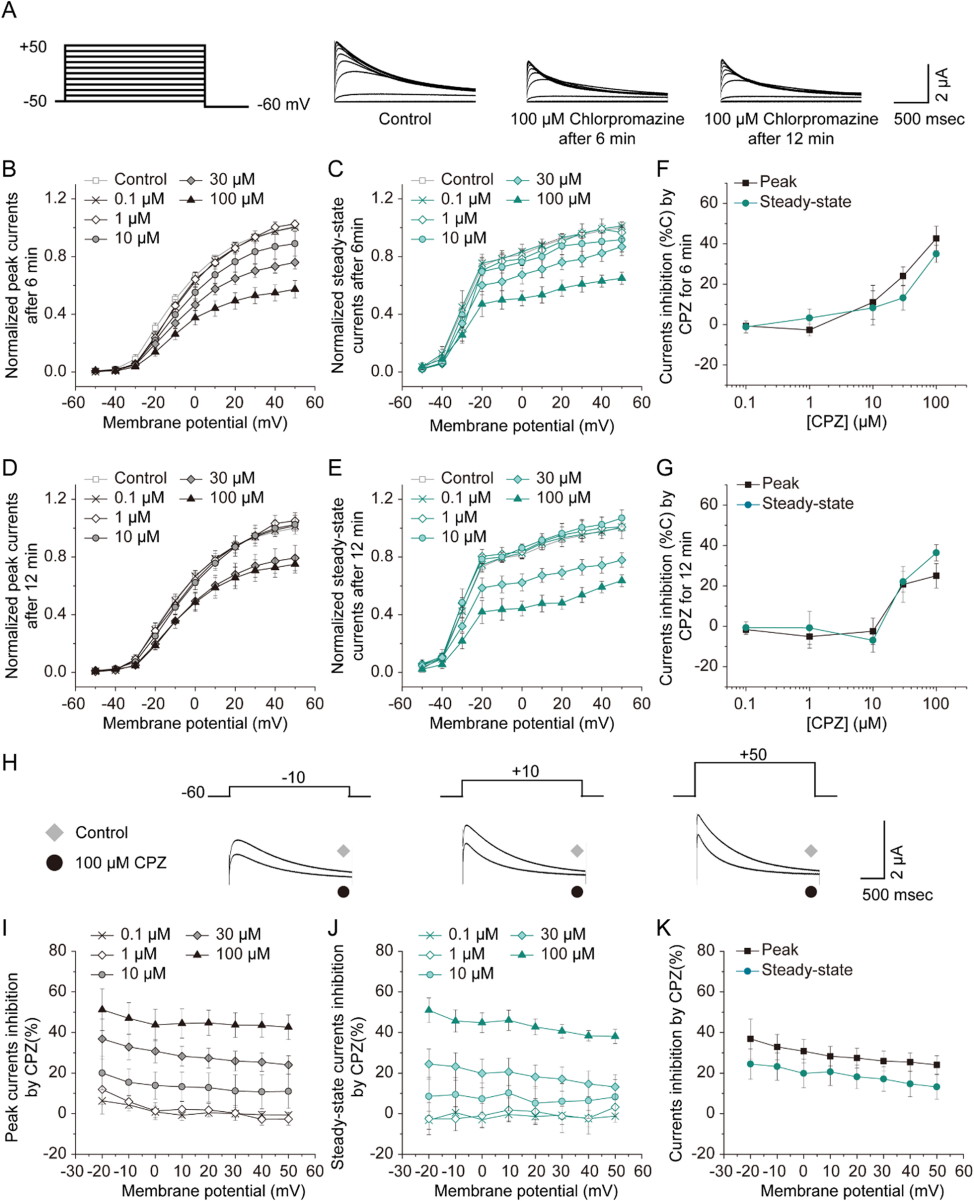
Direct inhibition of Kv1.3 channel currents by CPZ. **A** Superimposed current traces obtained by applying a series of voltage pulses from -50 mV to + 50 mV upon exposure to 100 µM CPZ for 6 and 12 min. **B**–**G** Current–voltage relationship of peak (**B**, **D**) and steady-state Kv1.3 channel currents (**C**, **E**) in the presence of CPZ with indicated concentrations for 6 min (**B**, **C**) or 12 min (**D**, **E**). Peak currents were recorded at their peaks, whereas steady-state currents were determined at the end of depolarizing pulses. Peak and steady-state currents at + 50 mV in control conditions were normalized to 1. Concentration–response inhibition of peak and steady-state currents after 6-min (**F**) or 12-min (**G**) exposure to CPZ, elicited by a single + 50 mV pulse from a holding potential of − 60 mV (n = 4–13 oocytes per concentration). **H** Current traces elicited by 2-s depolarization from − 20 to + 50 mV from a holding potential of − 60 mV, with and without 100 µM CPZ exposure. **I**, **J** CPZ-induced blockade of peak (**I**) and steady-state (**J**) Kv1.3 currents at various voltages for 6 min (n = 5–7 oocytes per treatment). At each depolarizing voltage step, the currents under different CPZ concentrations were normalized to the currents recorded without CPZ exposure. **K** Comparison of 30 µM CPZ-induced inhibition of peak and steady-state Kv1.3 currents at various voltages for 6 min. Current inhibition (%) = 100 × (current with vehicle—current with CPZ)/current with vehicle. Values are shown as mean ± S.E.M

We utilized a heterologous expression system of *Xenopus laevis* oocyte model [31]. We injected in vitro-transcribed complementary RNA (cRNA) encoding the human Kv1.3 channel into oocytes and performed two-electrode voltage clamp experiments with 100 μM CPZ, monitoring steady-state and peak currents. Our results indicate that CPZ suppressed both the peak and steady-state currents within 6 min (Fig. 1A). A concentration-dependent reduction in Kv1.3 channel currents was observed with CPZ concentrations ranging from 1 to 100 µM (Fig. 1B–G. The peak current at a + 50-mV depolarizing pulse was reduced to 57.4 ± 6.1% and 75.0 ± 6.0% of control levels after treatment with 100 µM CPZ for 6 and 12 min, respectively (n = 4–13; Fig. 1B, D). Similarly, the steady-state current was reduced to 65.0 ± 4.2% and 63.6 ± 4.1% of control levels after the same treatment durations, respectively (n = 5–13; Fig. 1C, E). These findings suggest that similar declines in peak and steady-state currents can be achieved within 6 min of CPZ exposure. Furthermore, CPZ showed a concentration-dependent inhibition of currents (Fig. 1F, G). The inhibition rates of peak and steady-state currents at 30 µM CPZ were 24.0 ± 4.5% and 13.2 ± 6.1% after 6 min (n = 5–13; Fig. 1F). At the same concentration, the inhibition rates for peak and steady-state currents were 20.7 ± 8.8% and 22.0 ± 5.1% after 12 min (n = 4–13; Fig. 1G). Hence, CPZ blocks Kv1.3 channels in a concentration-dependent manner, irrespective of the treatment duration (6 or 12 min). Comparing the effective concentration range of CPZ in Kv1.3-expressing *Xenopus* oocytes to its affinity for dopamine receptors poses challenges, as the presence of the vitelline membrane and yolk in oocytes may diminish the efficacy of CPZ [32]. These data suggest that CPZ acutely inhibits Kv1.3 channel currents directly, without involving other ion channels or receptors such as dopamine D2 receptors.

### Kv1.3 channel inhibition by CPZ is not voltage-dependent

The inhibition rate of Kv1.3 currents induced by CPZ was compared at different potential levels to investigate voltage effects (Fig. 1H). During 6 min of 30 µM CPZ treatment, Kv1.3 peak currents were inhibited by 36.8 ± 9.6%, 32.9 ± 6.2%, 30.8 ± 5.8%, 28.3 ± 4.8%, 27.4 ± 4.9%, 25.9 ± 4.9%, 25.4 ± 4.6%, and 24.0 ± 4.6% of control, indicating that CPZ-induced inhibition of Kv1.3 peak currents was not voltage-dependent (n = 5–7; P > 0.05, Fig. 1I). This voltage-independent suppression of Kv1.3 steady-steady currents by CPZ was consistent across concentrations ranging from 0.1 to 100 µM (n = 5–7; P > 0.05, Fig. 1J) and was also similar at peak current. Additionally, CPZ-induced inhibition of Kv1.3 currents did not significantly differ between peak and steady-state currents at the same test voltage from -20 to + 50 mV across concentrations ranging from 0.1 to 100 µM (n = 5–7; P > 0.05, Fig. 1K), although data for 0.1-, 1-, 10-, and 100-µM concentrations were not shown.

### Effects of CPZ on time constants for Kv1.3 channel activation and inactivation

**Fig. 2:**
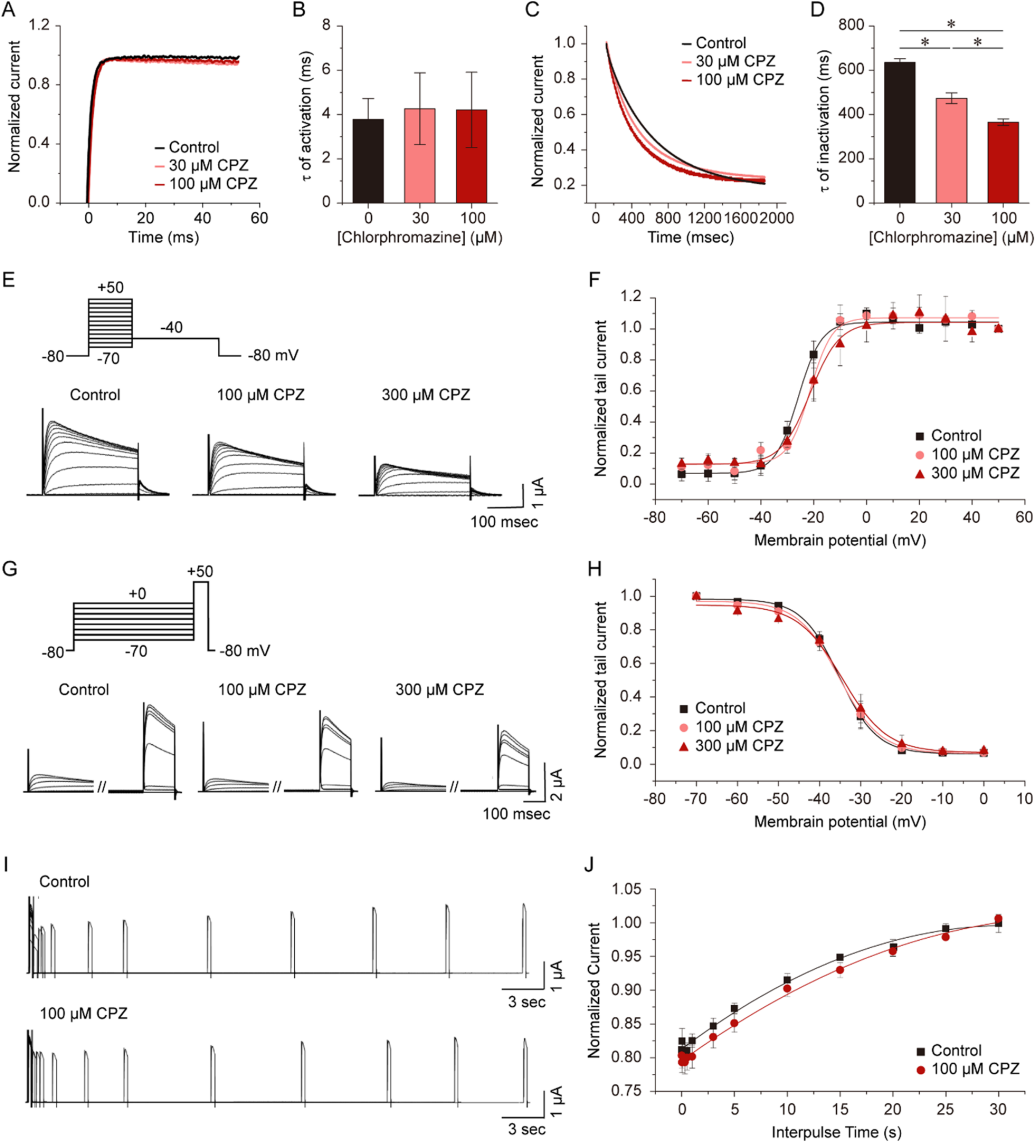
CPZ facilitates the inactivation of Kv1.3 Channel. **A**–**D** The activation and inactivation traces were fitted by single exponential functions, and the time constants of activation and inactivation processes were estimated from traces elicited by a single 2-s + 50 mV pulse from a holding potential of − 60 mV. **A** Representative normalized current traces of the activation phase in the absence and presence of 30 and 100 µM CPZ for 12 min. Each current trace was normalized to its peak value. **B** Time-constant values of activation processes with and without 30 and 100 µM CPZ (n = 5–7). **C** Representative normalized current traces of the inactivation phase in the absence and presence of 30 and 100 µM CPZ for 12 min. Each current trace was normalized to its peak value. **D** Time-constant values of inactivation processes with and without 30 and 100 µM CPZ (n = 9–10). **E** Typical steady-state activation tail currents measured at − 50 mV following 100-ms depolarizing pulses from − 70 to + 50 mV with and without 100 and 300 µM CPZ. **F** Steady-state activation curves obtained by normalizing each tail current to the tail current at + 50 mV and fitting the data to a Boltzmann equation (n = 6–7). **G** Typical tail currents evoked by 200-ms depolarizing pulses to + 40 mV; 30-s preconditioning pulses from − 70 to 0 mV with and without 100 and 300 µM CPZ. **H** Steady-state inactivation curves obtained by normalizing each tail current to the tail current when depolarized to + 40 mV and fitting the data to a Boltzmann equation (n = 7–8). *P < 0.05. **I** A double-pulse protocol was employed to investigate the recovery of Kv1.3 from inactivation, both in the absence and presence of CPZ. The first pulse consisted of a 200 ms depolarizing pulse to + 40 mV from a holding potential of − 80 mV, followed by a second identical pulse after varying interpulse intervals ranging from 10 ms to 30 s at − 80 mV. Pulses were delivered at intervals of 30 s. **J** The solid lines represent the single exponential fits of the peak amplitudes of Kv1.3 currents as a function of the interpulse interval (n = 4–5). Values are shown as mean ± S.E.M

The effect of CPZ on time constants for channel activation and inactivation was investigated (Fig. 2A–D). Exposure to 30 and 100 µM CPZ for 6 min resulted in activation time constants (τ) at + 50 mV of 3.8 ± 0.9, 4.3 ± 1.6, and 4.2 ± 1.7 ms, respectively (n = 5–7 oocytes, P < 0.05, Fig. 2A, B). These results indicate that CPZ at 30 and 100 μM for 12 min did not significantly change the activation time constant. Conversely, CPZ at 30 and 100 μM significantly decreased the inactivation time constants by the test pulse of + 50 mV to 473.7 ± 24.2 ms and 365.5 ± 14.7 ms, compared to the control value of 636.2 ± 16.6 ms (n = 9–10 oocytes, P < 0.05, Figs. 2C, D). The decay curves with the single exponential model are depicted in the Supplementary Fig. 1. Therefore, these results suggest that CPZ accelerates Kv1.3 channel inactivation, and the drug's effect on inactivation rate is greater than on activation.

### Effects of CPZ on steady-state Kv1.3 channel activation and inactivation

Two-pulse protocols were used to evoke tail currents and determine whether CPZ affects activation and inactivation gating. By fitting two different Boltzmann equations to normalized tail currents, steady-state activation and inactivation curves were obtained (Fig. 2E–H). The slope (k) of the activation curve was 4.4 ± 0.5, 4.1 ± 0.8, and 5.5 ± 0.9 for the control and CPZ (100 and 300 μM) groups, respectively (n = 6–7, P > 0.05, Fig. 2E, F). The *V*_*1/2*_ values of the activation curve for the control and CPZ (100 and 300 μM) groups were − 25.9 ± 0.6 mV, − 21.4 ± 0.8 mV, and − 21.4 ± 1.0 mV (n = 6–7, P > 0.05, Fig. 2F), respectively. For steady-state inactivation, the k values were 4.6 ± 0.2, 5.0 ± 0.4, and 5.4 ± 0.9 for the control and CPZ (100 and 300 μM) groups, respectively (n = 7–8, P > 0.05, Fig. 2G, H). The inactivation curve *V*_*1/2*_ values were − 35.2 ± 0.3 mV, − 35.3 ± 0.5 mV, and − 34.4 ± 1.0 mV for the control and CPZ (100 and 300 μM) groups, respectively (n = 7–8, P > 0.05, Fig. 2H). Thus, CPZ has no significant impact on the steady-state activation or inactivation kinetics at 100 and 300 μM, as indicated by the insignificant differences in *V*_*1/2*_ and k values.

### Effects of CPZ on the recovery from inactivation of Kv1.3 channel

Finally, we examination of whether CPZ alters the recovery from C-type inactivation of Kv1.3 channels. Recovery from the inhibition was investigated using a double-pulse protocol, with interpulse intervals ranging from 10 ms to 30 s (Fig. 2I–J). Under control conditions, the recovery from inactivation followed a single exponential function with a time constant of 12.98 ± 0.59 s (n = 5). Similarly, in the presence of 100 μM CPZ, the recovery process was best described by a single exponential function, yielding a time constant of 15.69 ± 1.14 s (n = 4). Notably, the time constant remained unchanged in the presence of CPZ. This observation suggests that the dissociation rate of CPZ is comparable to the transition rate between the open and closed states under control conditions, potentially explaining the use-independent inhibition induced by the drug.

## Discussion

In the previous study, we found that the anti-inflammatory effects of CPZ are due to the inhibition of Kv1.3 channels expressed in activated microglia of the mPFC [29]. In an animal model of LPS-induced systemic inflammation and microglial activation in vivo, we confirmed the upregulation of Kv1.3 in microglia of mPFC slices. We demonstrated that CPZ acutely blocked Kv1.3 currents and suppressed the LPS-induced upregulation of Kv1.3, suggesting that Kv1.3 is the primary microglial target of CPZ. However, this hypothesis has been challenged, particularly regarding (1) the potential involvement of dopamine receptors and (2) multiple cell types in the mPFC. Most of all, it was unclear whether it acts directly on microglial Kv1.3 channels or indirectly through other receptors, such as dopaminergic receptors, or other cell types like neurons or astrocytes. Microglia have been reported to express D2R in earlier studies, with observations made in primary microglia cultures [33, 34]. Upon brain ischemic injury, D2R expression was detected in microglia, indicating that injury settings can induce D2R expression [35, 36]. Additionally, CPZ has affinity for the D1 receptor [37]. While LPS did not alter D2 receptor expression, it did increase D1 receptor expression in microglia. Furthermore, dopamine receptor-mediated increases in pro-inflammatory cytokines have been reported in various contexts [38–42]. Therefore, it is necessary to consider the potential for indirect modulation of dopaminergic receptors, which could be CPZ targets in LPS-activated microglia. In our previous research we demonstrated that LPS enhances Kv1.3 channel expression, while CPZ effectively inhibits LPS-induced K^+^ conductance in mPFC microglia. Additionally, we showed that PAP-1, a Kv1.3 channel inhibitor, modulates the electrical properties and functions of microglia in a manner similar to CPZ. The comparable actions of PAP-1 and CPZ on mPFC microglia prompted us to hypothesize that CPZ mediates its effects via Kv1.3 channel inhibition.

In this study, we aimed to monitor the effect of CPZ on Kv1.3 channels without any possible involvement of other receptors or cell types. We found that CPZ directly inhibits the Kv1.3 channel in human Kv1.3 channel-expressing *Xenopus* oocytes. Additionally, we observed that CPZ may act on the Kv1.3 channel in a masking-block manner rather than as an open channel blocker. CPZ inhibited Kv1.3 currents in a voltage-independent manner and did not change the gating of either activation or inactivation. Moreover, the inhibition of Kv1.3 current by CPZ was similar for both peak and steady-state currents, indicating that CPZ interacts with both the open and closed states of the channel. Our findings indicate that CPZ does not affect the recovery process from C-type inactivation of the Kv1.3 channel. CPZ demonstrated no impact on the recovery time constant from inactivation, regardless of interval duration, suggesting its dissociation rate is comparable to the open-to-closed state transition. CPZ exhibits a unique characteristic differs from other Kv1.3 open channel blockers like rosiglitazone and troglitazone, which accelerate the decay rate of current inactivation in Kv1.3 channels [43]. Furthermore, compared to other K^+^ channels, CPZ inhibits the HERG channel in its open state without affecting activation or inactivation [32], suggesting distinct binding mechanisms across K^+^ channels. Rather than acting as a dopaminergic receptor inhibitor, these data suggest that CPZ functions as a Kv1.3 channel modulator. Kv1.3 channels have been reported to stabilize microglial membrane potential, thereby enhancing the driving force for Ca^2+^ influx and protecting the cells against rapid depolarization, which is essential for microglial activation [44]. Thus it can be anticipated that other drugs promoting accelerated inactivation of Kv1.3 channels may similarly inhibit the electrical properties and functions of microglia, akin to CPZ which is potentially linked to its immunosuppressive mechanism.

Regarding the pharmacological effects of CPZ in the mPFC, we cannot completely exclude indirect effects of neuronal dopamine receptor inhibition and altered neuron-glia communication [36]. Since CPZ in the mPFC targets various molecules other than microglial Kv1.3 channels, it may still exert indirect effects by acting on other molecules in different cell types. In addition, the anti-inflammatory effects of CPZ—which occur at micromolar concentrations—are likely achievable only at higher doses than those required for dopamine receptor antagonism. Careful consideration is required to establish the optimal dose range for exploring CPZ's anti-inflammatory effects through Kv1.3 channel inhibition, rather than its role as a dopamine receptor antagonist in clinical applications. Nevertheless, our results are significant in demonstrating that CPZ directly inhibits Kv1.3 without other assistance, providing a clearer understanding of its neuroinflammation-suppressing effect. Our study also contributes to elucidating the unknown effects of immunosuppressive drugs targeting the Kv1.3 channel.

Together, our results using the *Xenopus* oocyte heterologous expression system conclusively showed that CPZ accelerates the inactivation process and stabilizes the closed state of the Kv1.3 channel. These data validate that CPZ acts directly on the Kv1.3 channel, with Kv1.3 current inhibition independent of other receptors, including dopamine receptors. Our study aims to enhance the understanding of CPZ’s mechanism in inhibiting neuroinflammation and its effects on brain diseases by elucidating how CPZ directly inhibits Kv1.3 channels.

## Materials and methods

*Expression of Human Kv1.3 Channel in Xenopus Oocytes.* Complementary RNAs encoding the human Kv1.3 channel (GenBank accession no. BC035059.1) were synthesized by in vitro transcription using Message Machine T7 kits (Ambion, Austin, TX, USA) and stored in nuclease-free water at – 80 ℃. Stage V and VI oocytes were surgically harvested from female *Xenopus laevis* (Nasco, Modesto, CA, USA), anesthetized on ice for 30 min in 10-min intervals, and isolated from the theca and follicle layers using fine forceps. Two days later, the oocytes were injected with 20 nl of Kv1.3 cRNA (0.4 μg/μl). Injected oocytes were maintained at 17ºC in modified Barth’s solution containing 88 mM NaCl, 1 mM KCl, 0.4 mM CaCl_2_, 0.33 mM Ca(NO_3_)_2_, 1 mM MgSO_4_, 2.4 mM NaHCO_3_, 10 mM HEPES (pH 7.4), and 50 μg/ml gentamicin sulfate. Currents were measured 4–5 days after cRNA injection.

*Voltage Clamp Recording of Human Kv1.3 Channel in Xenopus Oocytes.* Kv1.3 channel-expressing oocytes were perfused with ND96 solution containing 96 mM NaCl, 2 mM KCl, 1.8 mM CaCl_2_, 1 mM MgCl_2_, and 10 mM HEPES (pH 7.4) at a constant flow rate in a bath chamber. Currents were measured at room temperature (20–23 °C) using a two-microelectrode voltage clamp system (OC-725C, Warner Instruments, Hamden, CT, USA). Both voltage and current-passing electrodes were filled with 3 M KCl. The voltage electrode resistance was 2.0–4.0 MΩ, and the current-passing electrode resistance was 2.0–2.5 MΩ. Stimulation and data acquisition were achieved using Digidata 1200 (Molecular Devices) and PClamp 9.2 (Molecular Devices). Kv1.3 channel currents were obtained by applying a series of voltage pulses from − 50 mV to + 50 mV of 2 s duration with 10 mV increments every 20 s from a holding potential of − 60 mV. The time constants of activation and inactivation processes were estimated using single exponential functions from current traces evoked by a single + 50 mV pulse of 2 s duration every 10 s from a holding potential of − 60 mV.

*Statistical Analysis*. All statistical analyses were performed using SPSS 26 software (IBM). Kv1.3 channel use dependency recordings were analyzed by repeated measures one-way analysis of variance (ANOVA) with post hoc Bonferroni or Friedman’s test. All data are presented as mean ± standard error of mean (SEM). A P < 0.05 was considered statistically significant.

## Supplementary Information

Supplementary Material 1.

## Data Availability

No datasets were generated or analysed during the current study.
