# Supplementary material for: Chlorpromazine directly inhibits Kv1.3 channels by facilitating the inactivation of channels
Source: Mol Brain. 2025 May 8;18:41. doi: 10.1186/s13041-025-01211-z (PMC12063219; doi:10.1186/s13041-025-01211-z)
Supplement: Supplementary file 1 — Supplementary Material 1. [file 13041_2025_1211_MOESM1_ESM.docx]

Supplementary Information for

Chlorpromazine directly inhibits Kv1.3 channels by facilitating the inactivation of channels

Seo-In Park^1,2,^*, Soobeen Hwang^2,^*, Young Lee^1,^*, Hee-Yoon Lee^1,^*, Soohyun Kim^1^, Junseo Hong^2^, Su-Hyun Jo^2^, Se-Young Choi^1^

^1^ Department of Physiology, Dental Research Institute, Seoul National University School of Dentistry, Seoul 03080, Republic of Korea.

^2^ Department of Physiology, Kangwon National University School of Medicine, Chuncheon 24341, Republic of Korea.

This PDF file includes:

Figure S1

**Supplementary Figure and Legends**


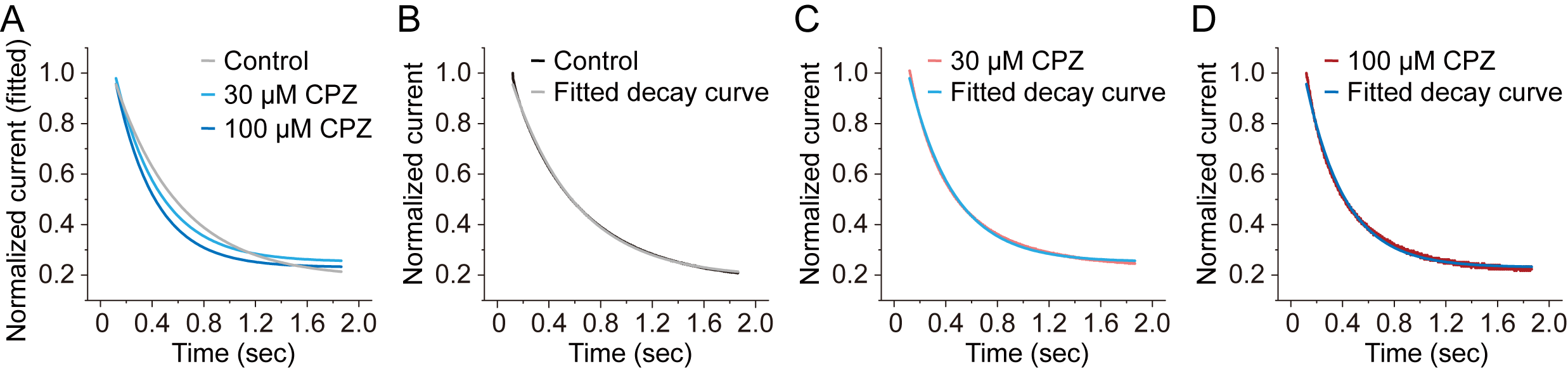


**Figure S1. The fitted decay curves with the single exponential model during the inactivation phase of Kv1.3 channel**

(A) Superimposed fitted decay curves of normalized current traces during the inactivation phase in the absence (light gray) and the presence of 30 µM CPZ (sky blue) and 100 µM CPZ (blue) over a 12-minute period.

(B) The normalized current trace (black, from Figure 2C) and its fitted decay curves (gray) during the inactivation phase in the absence of CPZ.

(C) The normalized current trace (pink, from Figure 2C) and its fitted decay curves (sky blue) during the inactivation phase in the presence of 30 µM CPZ.

(D) The normalized current trace (red, from Figure 2C) and its fitted decay curves (blue) during the inactivation phase in the presence of 100 µM CPZ.
